# The Red Back an Essential Medium

**Published:** 1904-05

**Authors:** 

**Affiliations:** Chicago


### The Red Back an Essential Medium.

CHICAGO, April 20, 1904.

*F. E. Daniel, M. D., Editor Texas Medical Journal, Austin, Texas.*

DEAR DOCTOR DANIEL: We take this occasion for thanking you for your kindly mention of Phospho-Albumen, in the current number of the TEXAS MEDICAL JOURNAL, and assure you of our appreciation of both the journal and your methods.

It may interest you to know that in making up our list for advertising, that the TEXAS MEDICAL JOURNAL has always appeared as an absolutely essential medium, as the writer has always appreciated its high standing and careful management. It has been our intention for some time to write you to this effect, but have neglected doing so.

Very sincerely,

THE PHOSPHO-ALBUMEN CO.

